# Racial and Ethnic Differences Among Active-Duty Service Members in Use of Mental Health Care and Perceived Mental Health Stigma: Results From the 2018 Health Related Behaviors Survey

**DOI:** 10.5888/pcd20.220419

**Published:** 2023-09-28

**Authors:** Lauren Piro, Huabin Luo, Katherine Jones, Suzanne Lazorick, Doyle M. Cummings, Sy Atezaz Saeed

**Affiliations:** 1 Healthcare Administrator, Navy Medicine Readiness and Training Command, New England, Newport, Rhode Island; 2 Department of Public Health, Brody School of Medicine, East Carolina University, Greenville, North Carolina; 3 Department of Psychiatry and Behavioral Medicine, Brody School of Medicine, East Carolina University, Greenville, North Carolina

## Abstract

**Introduction:**

The prevalence of mental health disorders is rising among US service members; however, research is limited on their use of mental health care. The objective of our study was to determine whether racial and ethnic disparities exist in the use of mental health care and perceived mental health stigma among active-duty service members.

**Methods:**

We obtained data from a sample of 17,166 active-duty service members who participated in the 2018 Department of Defense Health Related Behavior Survey (HRBS). Racial and ethnic groups included Black, Hispanic, White, and other. Yes–no questions about use of mental health care and perceived mental health stigma were our outcome variables. We used multiple logistic regression to assess racial and ethnic differences in mental health care use and perceived mental health stigma by service members. Significance was set at *P* <.*05.*

**Results:**

In 2018, approximately 25.5% of service members self-reported using mental health services, and 34.2% self-reported perceived mental health stigma. Hispanic service members (AOR = 0.78) and service members in the “other” racial and ethnic group (AOR = 0.81) were less likely than their White counterparts to have used mental health care. Black (AOR = 0.68) and Hispanic (AOR = 0.86) service members were less likely than their White counterparts to self-report perceived mental health stigma.

**Conclusion:**

The 2018 HRBS showed racial and ethnic differences in mental health care use and perceived stigma among US active-duty service members. Perceived stigma was a barrier to use of mental health care among service members with a mental health condition. Culture-sensitive programs customized for different racial and ethnic groups are needed to promote mental health care and reduce perceptions of stigma associated with its use.

**Summary**

**What is already known on this topic?**

Historically, US military service members have underutilized mental health services. A perceived stigma associated with mental health is a recognized barrier among service members to accessing mental health care.

**What is added by this report?**

Use of mental health care among service members and perception of a mental health stigma were last assessed in 2008. Our article uses data from the most recent national survey of service members to provide an update.

**What are the implications for public health practice?**

Differences exist by race and ethnicity in mental health care use and associated perceptions of stigma among active-duty US service members. More efforts are needed to improve access to mental health services and to address associated perceptions of stigma.

**MEDSCAPE CME**

In support of improving patient care, this activity has been planned and implemented by Medscape, LLC and *Preventing Chronic Disease*. Medscape, LLC is jointly accredited with commendation by the Accreditation Council for Continuing Medical Education (ACCME), the Accreditation Council for Pharmacy Education (ACPE), and the American Nurses Credentialing Center (ANCC), to provide continuing education for the healthcare team.

Medscape, LLC designates this Journal-based CME activity for a maximum of 1.0 AMA PRA Category 1 Credit(s)™. Physicians should claim only the credit commensurate with the extent of their participation in the activity.

Successful completion of this CME activity, which includes participation in the evaluation component, enables the participant to earn up to 1.0 MOC points in the American Board of Internal Medicine’s (ABIM) Maintenance of Certification (MOC) program. Participants will earn MOC points equivalent to the amount of CME credits claimed for the activity. It is the CME activity provider’s responsibility to submit participant completion information to ACCME for the purpose of granting ABIM MOC credit.

**Release date: September 28, 2023 Expiration date: September 28, 2024**

**Learning Objectives**

Upon completion of this activity, participants will:

1. Distinguish rates of mental health service use and perceived stigma against mental health disorders among US active-duty service members
2. Analyze variables among service members associated with a higher use rate of mental health services
3. Analyze variables among service members associated with higher perceived stigma against mental health disorders
4. Evaluate factors that might affect mental health service use among service members with mental health conditions

Credit Hours — 1.0

**EDITOR**

Rosemarie Perrin

Editor

*Preventing Chronic Disease*

Atlanta, Georgia

**AUTHORS**

Lauren Piro, MPH

Healthcare Administrator

Navy Medicine Readiness and Training Command, New England

Newport, Rhode Island

Huabin Luo, PhD

Department of Public Health

Brody School of Medicine

East Carolina University

Greenville, North Carolina

Katherine Jones, PhD

Department of Public Health

Brody School of Medicine

East Carolina University

Greenville, North Carolina

Suzanne Lazorick, MD, PhD

Department of Public Health

Brody School of Medicine

East Carolina University

Greenville, North Carolina

Doyle M. Cummings, PharmD

Department of Public Health

Brody School of Medicine

East Carolina University

Greenville, North Carolina

Sy Atezaz Saeed, MD, MS

Department of Psychiatry and Behavioral Medicine (Saeed)

Brody School of Medicine

East Carolina University

Greenville, North Carolina

**CME AUTHOR**

Charles P. Vega, MD

Health Sciences Clinical Professor of Family Medicine

University of California, Irvine School of Medicine

Charles P. Vega, MD, has the following relevant financial relationships:

Consultant or advisor for: Boehringer Ingelheim Pharmaceuticals, Inc.; GlaxoSmithKline; Johnson & Johnson

## Introduction

To ensure military readiness, service members must be capable of fulfilling all missions and tasks, including engaging in combat successfully and maximizing their deployment capability (1). Military service members must maintain good mental health in addition to physical strength and combat readiness (2). Service members who entered the military after September 11, 2001, are approximately twice as likely to serve in a combat deployment than those who entered before, putting them at a higher risk for emotional trauma (3). Improving mental health has become an important priority for the military (1,4–7).

Mental illness accounted for the most hospital days and was the second most common reason for a physician visit among active-duty service members in 2020 (8). A few studies have assessed mental health care use by veterans or by a single branch of the military (9–11), but research is limited on active-duty members. Quartana et al (4) evaluated trends in mental health care use and perceived stigma toward mental health (hereinafter, perceived stigma) among active-duty military from 2002 to 2011. However, their study focused only on the Army. Chu et al (5) examined mental health care use and perceived stigma among service members, but their analyses were based on 2008 Health Related Behavior Survey (HRBS) data. No studies have been conducted to assess mental health care use and mental health stigma among US service members since that study. During this time, many efforts have been made to improve mental health in the military. For example, in 2015 the Obama administration commissioned an evaluation of mental health public awareness campaigns to improve mental health in the military (12). However, data on the current status of perceived stigma associated with mental health and mental health care use among active-duty members are not available. Also, little research exists on racial and ethnic disparities in mental health care use. Currently, racial and ethnic minorities represent approximately 43% of active-duty service members, making the military more racially diverse today than in previous generations (13).

The main objectives of our study were to 1) examine racial and ethnic disparities in mental health care use and perceived mental health stigma among active-duty US service members by using the most recent Health Related Behaviors Survey (HRBS) data, and 2) assess the relationship between perceived stigma and mental health care use. Findings from this study may be used to inform policies and programs, to improve mental health education, and to increase use of mental health care in the military.

## Methods

Data used in this study were from the 2018 HRBS, which surveyed active and reserve components of the US military in the Air Force, Army, Marine Corps, Navy, and Coast Guard (14). HRBS is the US Department of Defense’s leading online survey used to gain an understanding of the health, health-related behaviors, and overall well-being of service members. It is conducted approximately every 3 years and uses a random sampling strategy stratified by service branch, pay grade, and sex. The 2018 HRBS was conducted by the RAND Corporation. The analytic weights were produced from the design and nonresponse weights, which were used to ensure that the final sample was representative of eligible service members from all 5 branches of the military. Missing data were addressed via multiple imputation by chained equations (MICE). A detailed introduction regarding the 2018 HRBS is available elsewhere (14). A total of 33,641 active duty and reserve service members participated in the 2018 HRBS. Given the objectives of our study, we focused on the active-duty service members (N = 17,166) who responded to the survey. Because our study used public data, it was exempt from institutional review board approval.

### Outcome variables

We assessed 2 binary outcomes in our analysis — self-reported mental health care use (yes/no) and perceived stigma (yes/no). The 2018 HRBS asked participants whether they had received any mental health care through either military or civilian services in the past year. We coded them as having received mental health care if they answered yes to the question, and no if they answered no. Participants were also asked whether they believed it would damage a person’s military career if they were to seek mental health counseling or treatment through the military (14). We coded them as having perceived stigma if they answered yes, or no if they answered no to the question. The perceived stigma variable was also included as a covariate in the model of mental health care use.

### Independent variable

The independent variable for our study was the self-reported racial or ethnic group. The 2018 HRBS obtained respondents’ race and ethnicity data from the military administrative records provided during the sampling process (14). Ethnicity (Hispanic or non-Hispanic) was coded first and took precedence. That is, respondents with Hispanic ethnicity were directly coded as Hispanic on the combined race and ethnicity variable in the data set, without any other accompanying race category. Non-Hispanic respondents were then coded based on their race (Black, White, or other). In the 2018 HRBS data set, race and ethnicity data were up-coded into 1 merged race and ethnicity variable — Black, Hispanic, White, and other.

### Covariates

To select covariates we used Andersen’s model of health services utilization (15–18), which has been used to describe differences in health care use. The model suggests that health service use is associated with predisposing, enabling, and need factors. In our analysis, predisposing factors included age (17–24 y, 25–34 y, 35–44 y, and ≥45 y), sex (male or female), marital status (married vs not married), and perceived stigma. Enabling factors included education (high school diploma or less, some college, and bachelor’s degree or more), service branch (Army, Air Force, Navy, Marine Corps, and Coast Guard), and military rank (enlisted or officer). Need factors included weight status based on body mass index (weight in kg/height in m^2^) reported as underweight or normal weight, overweight, or obese; self-reported medical conditions, reported as none, 1 to 2, or 3 or more; and the reported presence or absence (yes/no) of 1 of 8 mental health conditions (psychological distress, posttraumatic stress disorder [PTSD], or suicidal ideation). The 2018 HRBS defined psychological distress by the Kessler 6 Mental Health Scale (19) and PTSD by the Primary Care PTSD Screen for DSM-5 (20). Suicidal ideation was assessed with 1 survey question, which asked respondents whether they had had suicidal thoughts in the past 12 months.

### Statistical analysis

We first estimated the overall prevalence of the 2 outcome variables in 2018, use of mental health care and perceived stigma. We then used multiple logistic regression to assess racial and ethnic differences in these 2 outcome variables in 3 models. In Model 1, we controlled predisposing factors; in Model II, we added enabling factors to Model I. In Model III we added need factors to Model II. To assess whether odds ratios from the logistic regression models overestimated prevalence ratios, we repeated the analyses by using Poisson regression models with a robust error variance to calculate prevalence ratios (21). The perceived stigma variable was also included as a control variable (ie, a predisposing factor) in the mental health care use model. Analyses were conducted by using SAS 9.4 (SAS Institute Inc). Significance was set at *P* <.*05.*

## Results

### Descriptive results

The active duty service members, who made up the larger proportion of service members surveyed (N = 17,166) in 2018 HRBS, were aged 25 to 34 (39.9%), were male (83.3%), were White (58.0%), were enlisted service members (83.5%), were in the Army (34.5%), had a high school diploma or less (65.2%), were married (53.8%), were overweight (49.1%), and had no medical conditions (59.7%) (Table 1). Of active duty survey respondents, 25.5% (95% CI, 24.4%–26.6%) self-reported using mental health care services in the past year, and 34.2% (95% CI, 33.1%–35.4%) self-reported perceived stigma.

**Table 1:** Characteristics of US Active-Duty Military Participants (N = 17,166), 2018 Health Related Behaviors Survey^a^

Variables	Weighted % (95% CI)
**Age, y**	
17–24	37.8 (36.4–39.1)
25–34	39.9 (38.8–41.1)
35–44	18.3 (17.7–19.0)
≥45	4.0 (3.7–4.2)
**Sex**	
Male	83.3 (82.6–84.0)
Female	16.7 (16.0–17.4)
**Race or ethnicity**	
Black	16.3 (15.3–17.3)
Hispanic	16.1 (15.2–17.0)
White	58.0 (56.8–59.3)
Other	9.6 (8.9–10.2)
**Rank**	
Enlisted	83.5 (82.9–84.1)
Officer	16.5 (15.9–17.1)
**Service branch**	
Army	34.5 (33.2–35.8)
Air Force	24.1 (23.3–24.9)
Navy	24.4 (23.3–25.4)
Marine Corps	13.9 (13.1–14.7)
Coast Guard	3.2 (3.0–3.4)
**Education**	
High school diploma or less	65.2 (64.2–66.2)
Some college	13.0 (12.3–13.6)
Bachelor’s degree or less	21.9 (21.1–22.6)
**Marital status**	
Married	53.8 (52.6–55.1)
Not married	46.2 (44.9–47.4)
**Body mass index (weight in kg/height in m^2^)**	
Underweight or normal weight	36.6 (35.4–37.8)
Overweight	49.1 (47.8–50.3)
Obese	14.4 (13.5–15.2)
**Medical conditions**	
None	59.7 (58.5–60.9)
1–2	35.3 (34.2–36.5)
≥3	5.0 (4.5–5.4)
**Posttraumatic stress disorder**	
No	89.6 (88.9–90.4)
Yes	10.4 (9.6–11.1)
**Psychological distress**	
No	83.6 (82.6–84.5)
Yes	16.4 (15.5–17.4)
**Suicidal ideation**	
No	91.7 (91.0–92.5)
Yes	8.3 (7.5–9.0)
**Mental health care use**	
No	74.5 (73.5–75.6)
Yes	25.5 (24.4–26.6)
**Perceived stigma**	
No	65.8 (64.6–66.9)
Yes	34.2 (33.1–35.4)

a RAND Corporation. 2018 Health Related Behaviors Survey (14).

### Multiple regression model results

In the logistic regression model of mental health care use, Hispanic service members were less likely than White service members to use mental health care (AOR = 0.78; 95% CI, 0.65–0.94) (Table 2). Service members in the “other” racial or ethnic group were also less likely than White service members to use mental health care (AOR = 0.81; 95% CI, 0.66–0.99). The 3 mental health conditions — psychological distress, PTSD, and suicidal ideation — were significant correlates of mental health care use. Service members with these conditions were more likely than those without to use mental health care: psychological distress, AOR = 4.09 (95% CI, 3.39–4.93); PTSD, AOR = 2.47 (95% CI, 2.01–3.03); and suicidal ideation, AOR = 2.07 (95% CI, 1.59–2.71). Service members aged 35 to 44 years were more likely than those aged 17 to 24 years to use mental health care (AOR = 1.37; 95% CI, 1.12–1.68). Female service members were less likely than male service members to use mental health care (AOR = 0.59, 95% CI, 0.51–0.67) as were married versus unmarried service members (AOR = 0.78, 95% CI, 0.68–0.91). Service members with 1 to 2 medical conditions (AOR = 1.69, 95% CI, 1.46–1.94) and 3 or more medical conditions (AOR = 2.86; 95% CI, 2.25–3.63) were more likely than service members with no medical conditions to use mental health care. The perceived stigma variable was not significantly correlated with mental health care use (AOR = 0.98; 95% CI, 0.86–1.13; *P* >.05). Service members in the Air Force (AOR = 0.77; 95% CI; 0.66–0.89) and service members in the Coast Guard (AOR = 0.68; 95% CI, 0.56–0.84) were less likely than service members in the Army to use mental health care. Enlisted service members were more likely than officers to use mental health care (AOR = 1.22; 95% CI, 1.08–1.47).

**Table 2:** Logistic Regression Model Results of Factors Associated With Mental Health Care Use, Participants (N = 17,166), 2018 Health Related Behaviors Survey^a^

Variable	Prevalence of mental health care use, weighted % (95% CI)	Model I^b^	Model II^c^	Model III^d^
		AOR (95% CI)		
**Race**				
Black	28.1 (25.2–30.9)	1.07 (0.90–1.26)	0.98 (0.82–1.16)	0.95 (0.78–1.15)
Hispanic	22.5 (20.0–25.0)	0.83 (0.70–0.98)	0.77 (0.65–0.92)	0.78 (0.65–0.94)
White	25.7 (24.3–27.1)	1 [Reference]		
Other	23.8 (20.7–26.8)	0.86 (0.72–1.04)	0.83 (0.69–1.00)	0.81 (0.66–0.99)
**Age, y**				
17–24	24.7 (22.5–27.0)	1 [Reference]		
25–34	24.3 (22.8–25.8)	1.04 (0.89–1.22)	1.14 (0.96–1.34)	1.08 (0.90–1.30)
35–44	29.3 (27.8–30.7)	1.46 (1.24–1.71)	1.72 (1.44–2.04)	1.37 (1.12–1.68)
≥45	27.0 (24.3–29.6)	1.33 (1.09–1.62)	1.67 (1.34–2.08)	1.15 (0.89–1.49)
**Sex**				
Male	35.1 (33.2–37.0)	1 [Reference]		
Female	23.5 (22.3–24.8)	0.57 (0.51–0.64)	0.54 (0.48–0.60)	0.59 (0.51–0.67)
**Marital status**				
Not married	26.8 (25.0–28.6)	1 [Reference]		
Married	24.3 (23.1–25.6)	0.82 (0.72–0.94)	0.81 (0.72–0.93)	0.78 (0.68–0.91)
**Stigma**				
No	22.5 (21.2–23.8)	1 [Reference]		
Yes	31.3 (29.2–33.1)	1.60 (1.42–1.81)	1.60 (1.42–1.80)	0.98 (0.86–1.13)
**Education**				
High school diploma or less	25.8 (24.2–27.3)	—	1 [Reference]	
Some college	28.5 (26.2–30.7)	—	1.04 (0.89–1.20)	1.14 (0.97–1.33)
Bachelor’s degree or more	22.7 (21.4–24.0)	—	0.69 (0.61–0.78)	0.92 (0.81–1.05)
**Service branch**				
Army	28.7 (26.4–31.1)	—	1 [Reference]	
Air Force	20.3 (19.1–21.5)	—	0.61 (0.54–0.70)	0.77 (0.66–0.89)
Navy	27.0 (24.7–29.4)	—	0.87 (0.74–1.04)	0.86 (0.71–1.05)
Marine Corps	24.9 (22.4–27.4)	—	0.87 (0.72–1.06)	0.83 (0.67–1.03)
Coast Guard	19.3 (17.2–21.5)	—	0.54 (0.45–0.65)	0.68 (0.56–0.84)
**Rank**				
Officer	20.2 (18.8–21.6)	—	1 [Reference]	
Enlisted	26.5 (25.2–27.8)	—	1.54 (1.33–1.78)	1.22 (1.08–1.47)
**Psychological distress**				
No	18.6 (17.6–19.6)	—	—	1 [Reference]
Yes	60.6 (57.3–63.9)	—	—	4.09 (3.39–4.93)
**Posttraumatic stress disorder**				
No	21.5 (20.4–22.6)	—	—	1 [Reference]
Yes	59.7 (56.0–63.4)	—	—	2.47 (2.01–3.03)
**Suicidal ideation**				
No	22.1 (21.1–23.1)	—	—	1 [Reference]
Yes	62.9 (58.2–67.6)	—	—	2.07 (1.59–2.71)
**Body mass index (weight in kg/height in m^2^)**				
Underweight or normal weight	24.3 (22.4–26.3)	—	—	1 [Reference]
Overweight	24.2 (22.8–25.7)	—	—	1.00 (0.86–1.17)
Obese	32.5 (29.7–35.4)	—	—	1.21 (0.98–1.48)
**Medical conditions**				
None	19.1 (17.7–20.4)	—	—	1 [Reference]
1–2	32.8 (30.9–34.7)	—	—	1.69 (1.46–1.94)
≥3	50.1 (45.6–54.5)	—	—	2.86 (2.25–3.63)

Abbreviations: —, not applicable; AOR, adjusted odds ratio.

a RAND Corporation. 2018 Health Related Behaviors Survey (14).

b Model I: Race plus predisposing factors (age, sex, marital status, perceived stigma).

c Model II: Model I factors plus enabling factors (education, service branch, and military rank).

d Model III: Model II factors plus need factors (body mass index, self-reported medical conditions, and mental health conditions).

Black service members (AOR = 0.68, 95% CI, 0.58–0.81) and Hispanic service members (AOR = 0.86, 95% CI, 0.74–0.99) were less likely to report perceived stigma (Table 3). As in the mental health care use model, the 3 mental health condition variables were significant: service members with psychological distress (AOR = 2.12; 95% CI, 1.79–2.53), with PTSD (AOR = 1.67; 95% CI, 1.39–2.00), and with suicidal ideation (AOR = 1.99; 95% CI, 1.56–2.55) were more likely than those without to report perceived stigma.

**Table 3:** Logistic Regression Model Results of Factors Associated With Perceived Mental Health Stigma, Participants (N = 17,166), 2018 Health Related Behaviors Survey^a^

Variables	Prevalence of perceived stigma, weighted % (95% CI)	Model I^b^	Model II^c^	Model III^d^
		AOR (95% CI)		
**Race**				
Black	28.4 (25.5–31.3)	0.69 (0.59–0.81)	0.69 (0.59–0.81)	0.68 (0.58–0.81)
Hispanic	32.6 (29.7–35.5)	0.83 (0.72–0.97)	0.83 (0.72–0.97)	0.86 (0.74–0.99)
White	36.3 (34.8–37.8)	1 [Reference]		
Other	34.3 (30.8–37.8)	0.91 (0.76–1.08)	0.89 (0.75–1.05)	0.90 (0.75–1.07)
**Age, y**				
17–24	35.0 (32.6–37.4)	1 [Reference]		
25–34	34.6 (33.0–36.3)	1.00 (0.87–1.15)	0.98 (0.85–1.14)	0.95 (0.82–1.11)
35–44	33.2 (31.7–34.7)	0.93 (0.81–1.08)	0.89 (0.76–1.05)	0.77 (0.65–0.92)
≥45	27.8 (25.1–30.5)	0.70 (0.58–0.84)	0.65 (0.53–0.80)	0.54 (0.43–0.67)
**Sex**				
Male	35.1 (33.2–37.0)	1 [Reference]		
Female	34.1 (32.7–35.4)	0.93 (0.84–1.03)	0.93 (0.84–1.04)	1.04 (0.93–1.17)
**Marital status**				
Not married	34.6 (32.6–36.5)	1 [Reference]		
Married	34.0 (32.6–35.4)	0.99 (0.88–1.12)	1.00 (0.89–1.12)	1.00 (0.89–1.13)
**Education**				
High school diploma or less	34.1 (32.5–35.8)	—	1 [Reference]	
Some college	34.5 (32.1–36.9)	—	1.12 (0.97–1.29)	1.17 (1.01–1.35)
Bachelor’s degree or more	34.5 (33.0–36.0)	—	1.11 (0.99–1.24)	1.33 (1.18–1.49)
**Service branch**				
Army	33.0 (30.6–35.4)	—	1 [Reference]	
Air Force	32.2 (30.8–33.6)	—	0.92 (0.81–1.05)	1.08 (0.95–1.22)
Navy	37.3 (34.7–39.8)	—	1.19 (1.02–1.40)	1.20 (1.01–1.42)
Marine Corps	36.1 (33.3–38.9)	—	1.13 (0.94–1.34)	1.12 (0.94–1.34)
Coast Guard	31.3 (28.6–33.9)	—	0.89 (0.75–1.05)	1.06 (0.89–1.25)
**Rank**				
Officer	36.1 (34.4–37.8)	—	1 [Reference]	
Enlisted	33.9 (32.5–35.2)	—	0.87 (0.76–1.01)	0.77 (0.67–0.89)
**Psychological distress**				
No	29.8 (28.6–31.0)	—	—	1 [Reference]
Yes	56.7 (53.3–60.1)	—	—	2.12 (1.79–2.53)
**Posttraumatic stress disorder**				
No	31.9 (30.7–33.1)	—	—	1 [Reference]
Yes	54.3 (50.6–58.0)	—	—	1.67 (1.39–2.00)
**Suicidal ideation**				
No	31.7 (30.6–32.9)	—	—	1 [Reference]
Yes	62.0 (57.2–66.9)	—	—	1.99 (1.56–2.55)
**Body mass index (weight in kg/height in m^2^)**				
Under or normal	33.5 (31.4–35.5)	—	—	1 [Reference]
Overweight	33.7 (32.1–35.3)	—	—	0.99 (0.87–1.12)
Obese	38.0 (35.1–41.0)	—	—	1.10 (0.92–1.31)
**Medical conditions**				
None	30.4 (28.9–31.9)	—	—	1 [Reference]
1–2	39.4 (37.4–41.3)	—	—	1.37 (1.22–1.54)
≥3	43.4 (38.9–47.9)	—	—	1.50 (1.19–1.89)

Abbreviations: —, not applicable; AOR, adjusted odds ratio.

a RAND Corporation. 2018 Health Related Behaviors Survey (14).

b Model I: Race plus predisposing factors (age, sex, marital status, perceived stigma).

c Model II: Model I factors plus enabling factors (education, service branch, and military rank).

d Model III: Model II factors plus need factors (body mass index, self-reported medical conditions, and mental health conditions).

Service members aged 35 to 44 (AOR = 0.77; 95% CI, 0.65 to 0.92) and 45 years or older (AOR = 0.54; 95% CI, 0.43–0.67) were less likely than those aged 17 to 24 years to report perceived stigma. Service members with some college (AOR=1.17; 95% CI, 1.01–1.35) or a bachelor’s degree or more (AOR = 1.33; 95% CI, 1.18–1.49) were more likely to report perceived stigma than those with a high school diploma or less. Service members with 1 to 2 medical conditions (AOR = 1.37; 95% CI, 1.22–1.54) or 3 or more medical conditions (AOR = 1.50; 95% CI, 1.19–1.89) were more likely than those with no medical conditions to report perceived stigma. Navy service members were more likely than Army service members to report perceived stigma (AOR = 1.20; 95% CI; 1.01–1.42). Enlisted service members were less likely than officers to report perceived stigma (AOR = 0.77; 95% CI, 0.67–0.89) (Table 3).

We ran Poisson regression models for mental health care use and 1 model for perceived stigma to assess the race and ethnicity variable. For mental health care use, the prevalence ratio (PR) for Black service members compared with White service members was 0.96 (95% CI, 0.85–1.07); for Hispanic service members, 0.86 (95% CI, 0.76–0.97); and for service members of other racial or ethnic groups, 0.89 (95% CI, 0.78–1.00). For perceived stigma, the PR for Black service members compared with White service members was 0.79 (95% CI, 0.71–0.88); for Hispanic service members, 0.91 (95% CI, 0.83–1.00); and for service members of other racial or ethnic groups, 0.93 (95% CI, 0.84–1.04).

In stratified analyses of participants with and without a mental health condition (ie, whether service members reported psychological distress, PTSD, or suicidal ideation), race or ethnicity was not a significant factor among those with a mental health condition. However, the perceived stigma variable was significant. Service members who self-reported perceived stigma were less likely to use mental health care (AOR = 0.72; 95% CI, 0.56–0.92) (Table 4). In the sample without a mental health condition, Hispanic service members were less likely to use mental health care (AOR = 0.79; 95% CI, 0.63–0.98). The perceived stigma variable was not significant.

**Table 4:** Logistic Regression Model Results of Factors Associated With Mental Health Care Use Among Service Members With (n = 3,597) and Without (n = 13,569) a Mental Health Condition, 2018 Health Related Behaviors Survey^a^

Variables	With a mental health condition	Without a mental health condition
	AOR (95% CI)	
**Race**		
White	1 [Reference]	
Black	1.05 (0.71–1.55)	0.92 (0.74–1.16)
Hispanic	0.79 (0.57–1.09)	0.79 (0.63–0.98)
Other	0.77 (0.54–1.09)	0.83 (0.65–1.05)
**Age, y**		
17–24	1 [Reference]	
25–34	1.26 (0.91–1.73)	1.00 (0.80–1.25)
35–44	1.55 (1.07–2.24)	1.29 (1.01–1.65)
≥ 45	2.17 (1.29–3.65)	0.91 (0.67–1.24)
**Sex**		
Male	1 [Reference]	
Female	0.63 (0.49–0.82)	0.56 (0.48–0.66)
**Marital status**		
Not married	1 [Reference]	
Married	0.87 (0.67–1.13)	0.75 (0.64–0.89)
**Stigma**		
No	1 [Reference]	
Yes	0.72 (0.56–0.92)	1.15 (0.98–1.35)
**Education**		
High diploma school or less	1 [Reference]	
Some college	1.11 (0.82–1.50)	1.14 (0.94–1.37)
Bachelor’s degree or more	1.06 (0.82–1.37)	0.90 (0.76–1.05)
**Service branch**		
Army	1 [Reference]	
Air Force	0.97 (0.73–1.30)	0.72 (0.60–0.86)
Navy	0.77 (0.55–1.10)	0.92 (0.73–1.16)
Marine Corps	0.86 (0.60–1.26)	0.82 (0.63–1.07)
Coast Guard	0.69 (0.45–1.05)	0.70 (0.56–0.89)
**Rank**		
Officer	1 [Reference]	
Enlisted	1.28 (0.94–1.74)	1.23 (1.02–1.49)
**Body mass index (weight in kg/height in m^2^)**		
Underweight or normal weight	1 [Reference]	
Overweight	0.99 (0.74–1.32)	1.02 (0.86–1.22)
Obese	1.26 (0.86–1.84)	1.17 (0.92–1.51)
**Medical conditions**		
None	1 [Reference]	
1–2	1.65 (1.26–2.15)	1.66 (1.41–1.96)
≥3	2.38 (1.54–3.67)	3.05 (2.31–4.03)

Abbreviation: AOR, adjusted odds ratio.

a RAND Corporation. 2018 Health Related Behaviors Survey (14). In both models (ie, the model for those with a mental health condition and the model for those without a mental health condition), the independent variable was race, and covariates included predisposing factors (age, sex, marital status, and perceived stigma), enabling factors (education, service branch, and military rank), and need factors (body mass index, and self-reported medical conditions).

## Discussion

Analyzing 2018 HRBS data, the most recent available, we assessed racial and ethnic disparities in mental health care use and perceived mental health stigma, and the relationship between perceived stigma and mental health care use among active-duty US service members. Results from our multiple regression model show that Hispanic service members and service members of other racial and ethnic groups were less likely than White service members to use mental health care. The results also show that Black and Hispanic service members were less likely than White service members to report mental health stigma. In 2018, only about one-quarter of service members self-reported having used mental health care, and about one-third self-reported mental health stigma.

For mental health care use, our descriptive results (unadjusted prevalence) did not show a significant difference between White service members and others. But results in our multiple logistic regression model showed significant differences, a finding consistent with prior findings among military populations (5,22–24). In an analysis using 2008 HRBS data, Chu et al (5) found that minority service members were less likely to seek mental health care, and that Asian service members were the least likely of any racial or ethnic group. They also found that among service members with mental health conditions and perceived stigma, Asian service members were less likely to use mental health care than non-Asian service members. In another study that used data from the 2011 Army Study to Assess Risk and Resilience in Service Members, Colpe et al (22) found that non-Hispanic Black service members were less likely to use mental health care than White service members. Studies of the general population also found that racial and ethnic minorities were less likely to use mental health care (22,23). Cabassa et al (23) conducted a review of studies on Hispanic adults’ access to mental health care and found that they were less likely than White adults to use mental health care. And when Hispanic adults did seek care, it was usually from a general physician rather than a mental health specialist. In the general population, having health insurance was associated with a greater likelihood of seeking mental health care (22,23); however, health insurance should not be relevant for service members because health care is provided for them. Thus, other factors, such as easy access to mental health care providers, may improve use of mental health care. Significant differences in use across the different branches of the military are also of note, with Air Force and Coast Guard service members being less likely to use mental health care than service members in the Army. These findings deserve more attention. Limited access to mental health care providers (25,26) may account for the findings of lower use among service members in the Air Force and Coast Guard.

For perceived stigma, our descriptive results show a significant difference between White and Black service members. Our multiple logistic regression model showed that Black and Hispanic service members were less likely to report perceived stigma, which is consistent with prior findings. For instance, Skopp et al (11) found significant differences in perceived stigma between racial and ethnic groups, with White service members being more likely to self-report perceived stigma than Black service members. Our findings also showed that service members in the Navy were more likely to report perceived stigma about mental health care than those in the Army. The reason for these findings is not known. More efforts are needed to promote mental health care and eliminate prejudice against mental health issues.

An interesting finding from our study is that Hispanic service members were less likely to report perceived stigma but were still less likely to use mental health care. This suggests that there may be other barriers or underlying reasons for not seeking mental health care among this minority group, such as cultural expectations, wanting to be self-reliant, or limited knowledge about mental health (23). Cabassa et al (23) found that perceived stigma and low acculturation levels among Hispanic adults were negatively associated with the mental health care use. Differences among different racial and ethnic groups should be further investigated. Future studies should evaluate the possibility of unique barriers among minority groups to accessing and using mental health care.

In our analysis by mental health status, we found that perceived stigma was associated with decreased odds of seeking mental health care among service members with a mental health condition (ie, psychological distress, PTSD, or suicidal ideation). This finding is cause for concern. Similar findings were also reported in prior research. In a study using data from a survey of active-duty service members, Britt and colleagues (27) found that participants with a mental health condition had higher perceived stigma scores and were less likely to seek mental health treatment than those without. They suggested that the modified labeling theory of perceived stigma, which posits that a service member who has negative views of their peers who seek mental health treatment is likely to internalize these perceptions, may account for this. This is especially problematic when that service member develops a mental health problem of their own but delays seeking treatment because of internalized negative feelings.

Many studies have examined the association between perceived stigma and mental health care use (11,27–29), but findings are mixed. Some studies found that perceived stigma was associated with less mental health care use (6,7,30,31). Other studies found that perceived stigma increased mental health care use (32). The different samples and study designs may account for these different findings across studies. The association between perceived stigma and mental health care use should be closely monitored.

In our study, about 25% of service members self-reported having used mental health care in 2018, and about 34% self-reported perceived stigma. Quartana et al (4) found that in 2008, 28.2% of Army soldiers used mental health care, and 35.3% self-reported perceived stigma. Their study also found a consistent decline in perceived stigma, from 48% in 2002 to 35.3% in 2008. However, their study sample was from a single branch of service, whereas our study included all 5 branches (Army, Navy, Marine Corps, Air Force, and Coast Guard). Although that study is not directly comparable with ours, taken together, our findings and prior findings suggest that the prevalence of perceived stigma remained high and was unchanged overall from 2008 to 2018. Perceived stigma is an ongoing concern, and more efforts are needed to understand and address it.

On a positive note, recent programs, such as the 2009 Real Warriors Campaign (which aimed to break down mental health stigma) and the inTransition Program (a free confidential program that provides access to mental health care) (33), have been implemented to reduce perceived stigma toward using mental health care. The Organizational Incident Operational Nexus Trauma Tracker (34), started in 2017, aimed to provide long-term mental health support to sailors who had been exposed to potentially traumatic events. Sailors are contacted by telephone or email at their request to connect them to mental health services. These programs advocate for the importance of tracking and improving mental health among service members. Because distance and work schedule may be a barrier to seeking mental health care, mobile mental health clinics and telehealth-delivered care should also be considered as methods to increase use (35).

Our study had limitations. First, HRBS data were self-reported and are subject to bias. Second, the measure of mental health care use was binary, and we did not account for the frequency or intensity of mental health treatment service members received. The measure of perceived stigma was also binary. Third, no data on Asian service members were available. These service members are the third largest racial and ethnic group in the military and the second largest minority group (36). Future surveys should include more Asian and other minority service members. Fourth, in our data set, race and ethnicity were combined into one variable. We could not separate respondents by Hispanic ethnicity.

### Conclusion

The 2018 HRBS data showed racial and ethnic differences in use of mental health care and perceived stigma among US active-duty service members. Perceived stigma was a barrier to mental health care use among service members with a mental health condition. Continued efforts are needed in the US military to promote mental health awareness, improve access, and address mental health stigma. Culture-sensitive programs customized for different racial and ethnic groups are needed to promote mental health care and reduce any stigma associated with its use.

## Post-Test Information

To obtain credit, you should first read the journal article. After reading the article, you should be able to answer the following, related, multiple-choice questions. To complete the questions (with a minimum 75% passing score) and earn continuing medical education (CME) credit, please go to http://www.medscape.org/journal/pcd. Credit cannot be obtained for tests completed on paper, although you may use the worksheet below to keep a record of your answers.

You must be a registered user on http://www.medscape.org. If you are not registered on http://www.medscape.org, please click on the “Register” link on the right hand side of the website.

Only one answer is correct for each question. Once you successfully answer all post-test questions, you will be able to view and/or print your certificate. For questions regarding this activity, contact the accredited provider, CME@medscape.net. For technical assistance, contact CME@medscape.net. American Medical Association’s Physician’s Recognition Award (AMA PRA) credits are accepted in the US as evidence of participation in CME activities. For further information on this award, please go to https://www.ama-assn.org. The AMA has determined that physicians not licensed in the US who participate in this CME activity are eligible for AMA PRA Category 1 Credits™. Through agreements that the AMA has made with agencies in some countries, AMA PRA credit may be acceptable as evidence of participation in CME activities. If you are not licensed in the US, please complete the questions online, print the AMA PRA CME credit certificate, and present it to your national medical association for review.

## Post-Test Questions

### Study Title: Perceived Stigma Against Mental Health Disorders and Mental Health Service Utilization Among US Active-Duty Service Members

#### CME Questions

1. What were the approximate rates of use of mental health services and perceived mental health stigma, respectively, in the current study?
  - 10% and 70%
  - 20% and 50%
  - 25% and 35%
  - 45% and 20%
2. Which one of the following variables was associated with a higher rate of use of mental health services in the current study?
  - Hispanic people or people of other race/ethnicity
  - The presence of posttraumatic stress disorder (PTSD)
  - Age between 17 and 24 years
  - Less perceived mental health stigma
3. Which of the following variables was associated with a higher rate of perception of stigma against mental health disorders in the current study?
  - Black race and Hispanic ethnicity
  - The presence of psychological distress
  - Age 45 years and older
  - Lower educational attainment
4. In the analysis limited to service members with a mental health condition in the current study, which one of the following variables was most significant in determining their use of mental health services?
  - Black race
  - White race
  - Marital status
  - Perceived stigma against mental health disorders
